# Transmission dynamics and molecular characterization of methicillin-resistant *S. epidermidis* among men who have sex with men in Guangzhou, China

**DOI:** 10.1128/spectrum.00792-25

**Published:** 2025-11-06

**Authors:** Sitong Chen, Yuxia Wu, Yuguo Liu, Qi Cheng, Zhenjiang Yao, Xiaohua Ye

**Affiliations:** 1 School of Public Health, Guangdong Pharmaceutical University 71237, Guangzhou, China; University of Pretoria, Pretoria, Gauteng, South Africa

**Keywords:** Methicillin-resistant* Staphylococcus epidermidis*, men who have sex with men, antimicrobial resistance, transmission dynamics, genomic epidemiology

## Abstract

Methicillin-resistant *Staphylococcus epidermidis* (MRSE) is a major opportunistic pathogen associated with high morbidity and mortality. However, its transmission dynamics and molecular characteristics in high-risk populations such as men who have sex with men (MSM) remain poorly understood. A community-based cross-sectional study was performed to recruit 510 MSM, providing nasal swabs for MRSE analysis. All MRSE isolates were characterized using antimicrobial susceptibility testing, whole-genome sequencing, and phylogenetic analysis, so as to reveal genomic diversity and transmission clusters. Among 510 MSM participants, the nasal colonization rates of *S. epidermidis* and MRSE were 82.75% and 44.12%, respectively. Genomic analyses of 190 MRSE isolates identified 20.53% as homologous, forming 12 transmission clusters and 40 transmission routes. In addition, homologous isolates exhibited distinct resistance and virulence profiles (*tet(K*), *ANT(4′)-Ib, esaA*, *esaB*, *icaA*, *icaB*, *icaC*, *icaR*, *essA*, *essB*, *essC*, and *esxA*; all *P* < 0.05) and genotypes (ST130, ST20, ST35, and ST57; all *P* < 0.05). The final Random Forest model achieved an accuracy of 81.58% in predicting homologous transmission risk. These findings highlight the high carriage risk and complex dynamics of MRSE transmission in this high-risk population, emphasizing the need for targeted surveillance and infection control strategies.

**IMPORTANCE:**

This study uncovers high nasal methicillin-resistant *Staphylococcus epidermidis* (MRSE) colonization (44.12%) and complex transmission dynamics among men who have sex with men (MSM), a key reservoir for multidrug-resistant *S. epidermidis*. Whole-genome analysis identified 12 transmission clusters and 40 transmission routes, with homologous isolates enriched in *tet(K*) and *ANT(4′)-Ib* resistance genes but lacking biofilm-associated *ica* genes. A Random Forest model achieved a classification accuracy of 81.58% for predicting transmission risk. These findings highlight MSM as critical hubs for community MRSE spread and provide actionable targets for surveillance, guiding effective infection control strategies to curb antimicrobial resistance.

## INTRODUCTION

*Staphylococcus epidermidis* (*S. epidermidis*) is a ubiquitous commensal bacterium that asymptomatically colonizes the human skin and mucous membranes (1). It is recognized as an important opportunistic pathogen that poses serious challenges when breaching the epithelial barrier, especially for nosocomial infections associated with invasive procedures and indwelling medical devices (2). The extensive use of antibiotics has led to *S. epidermidis* developing resistance to various antibiotics (3). The 2022 National Antimicrobial Resistance Surveillance Report (4) revealed that the detection rate of *S. epidermidis* and methicillin-resistant *S. epidermidis* (MRSE) in intensive care units in China significantly surpassed that found in other wards and also suggested that *S. epidermidis* is the predominant coagulase-negative *Staphylococcus* found in hospital wards. Except for the ubiquity in hospital settings, it was also prevalent in a variety of community settings. A study in Iran revealed the nasal carriage rate of *S. epidermidis* among healthcare workers was high, at 42%, and similar high carriage rates were observed in the community population in Portugal (54%) and China (44.8%) (5–7). These studies highlight the potential for healthy individuals acting as high-risk carriers of this pathogen. Methicillin as the traditional antibiotic for treating staphylococcal infections has become less effective due to the increasing emergence of methicillin-resistant strains. In many countries, 75%–90% of hospital-associated *S. epidermidis* isolates are methicillin-resistant, which is significantly higher than that of *Staphylococcus aureus* (40%–60%) (8). In the Netherlands, the effective implementation of “search-and-destroy” programs coupled with strict hygiene protocols has significantly decreased the prevalence of methicillin-resistant *S. aureus* in hospital settings (9). However, these interventions have shown less efficacy against MRSE (10). Notably, MRSE isolates are more frequently isolated from pathogens in clinical samples compared to methicillin-sensitive isolates (11). Therefore, there is a pressing need to monitor this pathogen in hospital and community settings.

Men who have sex with men (MSM) may represent a critical reservoir for MRSE transmission due to unique behaviors and immunological vulnerabilities. Different from the general community population, MSM maintain complex networks of sexual partners, characterized by high partner turnover rates and various risky behaviors (e.g., caressing, masturbation, unprotected oral, and anal sex), which may increase potential transmission risk of various pathogens (12). Considering the unique physiological and behavioral features, the phenotypic and genotypic characteristics of MRSE isolates from MSM may be different from general community populations. For example, frequent anal intercourse may lead to increased damage to the rectal mucosa, which in turn increases the risk of infection (13). Moreover, there is an outbreak trend of certain pathogenic bacteria from the MSM population in Germany and America, which are not only risk factors for various diseases but also have potential for exacerbating the health burden among individuals with HIV infection (14–16). However, systematic surveillance data regarding MRSE colonization dynamics and transmission patterns within MSM populations remain conspicuously scarce. Defining the homologous transmission isolates is the first and most important step for identifying and preventing transmission risk of MRSE, but the specific biomarkers that distinguish between homologous and non-homologous MRSE isolates are still unclear.

To combat this opportunistic pathogen, there is a pressing need to monitor transmission dynamics of MRSE and identify the super-spreading genotypes. Therefore, we performed homology analyses of MRSE isolates from MSM to identify homologous transmission clusters. In addition, we compared the genomic differences between homologous and non-homologous isolates, aiming to identify high-risk genotypes. Our findings may inform targeted surveillance strategies to prevent MRSE dissemination in vulnerable populations.

## RESULTS

### Prevalence of nasal colonization with *S. epidermidis* and MRSE

Among 510 MSM in this study, we identified 422 *S*. *epidermidis* isolates, with a nasal colonization rate of 82.75% (422/510). Additionally, we identified 225 MRSE isolates, corresponding to a nasal colonization rate of 44.12% (225/510). Whole-genome sequencing was successfully performed on 190 MRSE isolates. According to the homology threshold (with a single nucleotide polymorphism [SNP] distance of less than 71 between two isolates defined as homologous isolates), we identified 20.53% (39/190) as homologous isolates and 79.47% (151/190) as non-homologous isolates.

### Antimicrobial resistance of homologous and non-homologous MRSE isolates

As shown in Table 1, all MRSE isolates were susceptible to vancomycin and rifampicin, and most of MRSE isolates were susceptible to linezolid, chloramphenicol, and gentamicin. However, most of the MRSE isolates were resistant to penicillin (93.68%), followed by cefoxitin (85.26%) and erythromycin (75.79%). In the homologous isolates, the predominant resistance pattern was penicillin-erythromycin-clindamycin-tetracycline-cefoxitin, accounting for 17.9%. In the non-homologous isolates, the most common resistance pattern was penicillin-erythromycin-cefoxitin, accounting for 5.2%. When comparing the antibiotic resistance between homologous and non-homologous isolates (Table 1), we observed that homologous MRSE isolates had significantly lower rates of resistance to erythromycin (61.54% vs 79.47%, *P* = 0.020) and chloramphenicol (2.56% vs 16.00%, *P* = 0.017) than non-homologous isolates.

**TABLE 1:** Antimicrobial resistance of homologous and non-homologous MRSE isolates^*b*^^,^^*c*^

Antimicrobial resistance	MRSE isolates (*n* = 190)	Homologous isolates (*n* = 39)	Non-homologous isolates (*n* = 151)	*P-*value
Penicillin	178 (93.68)	38 (97.44)	140 (92.72)	0.280
Cefoxitin	162 (85.26)	36 (92.31)	126 (83.44)	0.164
Erythromycin	144 (75.79)	24 (61.54)	120 (79.47)	**0.020**
Clindamycin	100 (52.63)	23 (58.97)	77 (50.99)	0.374
Tetracycline	75 (39.47)	17 (43.59)	58 (38.41)	0.555
Cotrimoxazole	68 (35.79)	12 (30.77)	56 (37.09)	0.463
Teicoplanin	66 (34.74)	11 (28.21)	55 (36.42)	0.337
Levofloxacin	56 (29.47)	8 (20.51)	48 (31.79)	0.169
Gentamicin	27 (14.21)	4 (10.26)	23 (15.23)	0.428
Chloramphenicol	25 (13.16)	1 (2.56)	24 (16.00)	**0.017** ^*a*^
Linezolid	7 (3.68)	3 (7.69)	4 (2.65)	0.136
Rifampicin	0 (0.00)	0 (0.00)	0 (0.00)	–^*d*^
Vancomycin	0 (0.00)	0 (0.00)	0 (0.00)	–

^*a*^ Estimated by the Fisher’s exact probability method.

^*b*^ Data are presented as no. (%) or as otherwise indicated. Boldface indicates statistical significance.

^*c*^ MRSE, methicillin-resistant *S. epidermidis.*

^*d*^ –, not applicable.

### Molecular characteristics of homologous and non-homologous MRSE isolates

**Fig 1:**
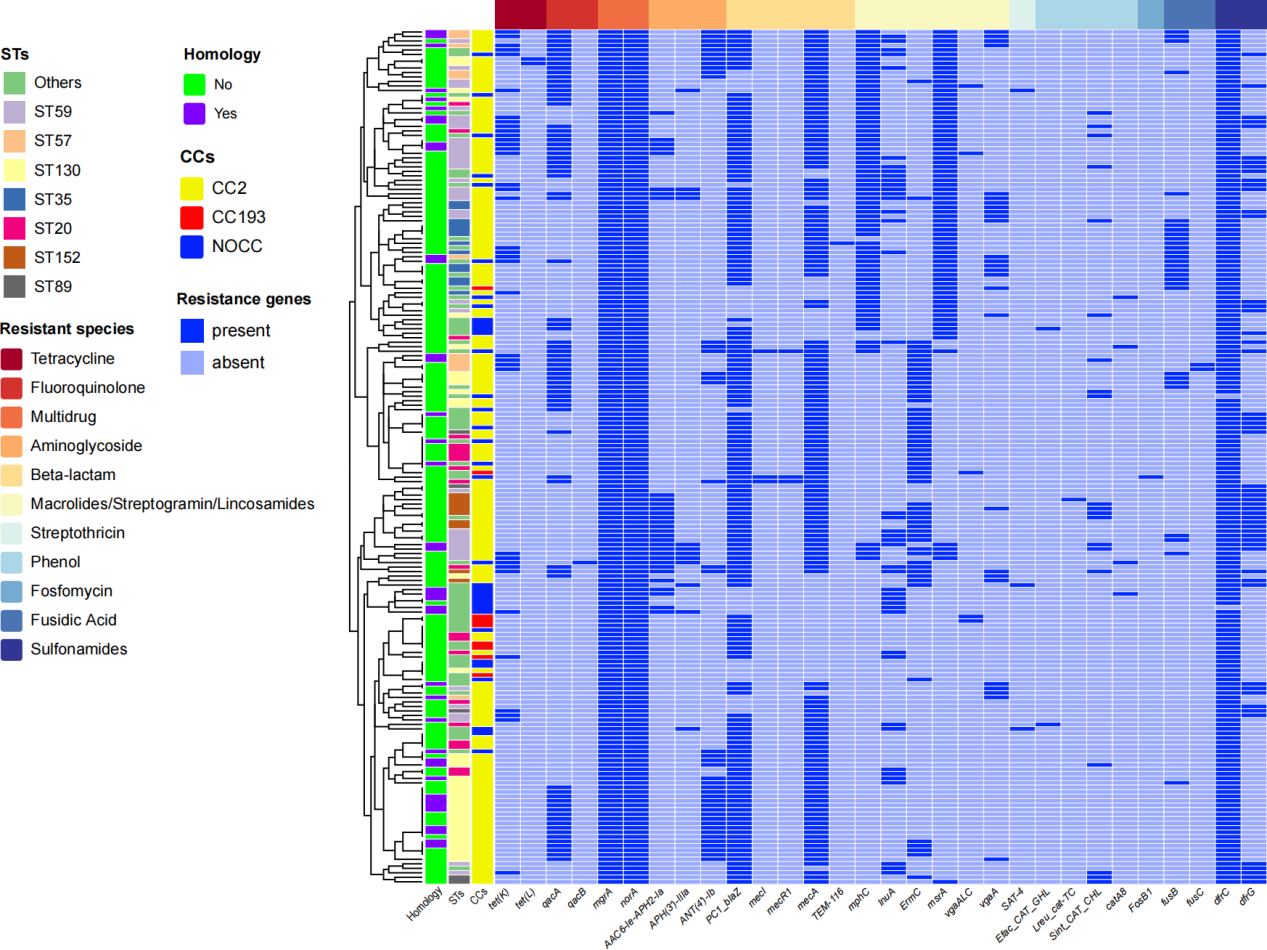
Antimicrobial resistance genes of homologous and non-homologous MRSE isolates.

Among 190 MRSE isolates, a total of 31 genes for resistance were identified, and each isolate carried a minimum of three and a maximum of 14 genes for resistance (Fig. 1). Notably, two multidrug-resistant genes (*mgrA* and *norA*) were detected in all MRSE isolates, followed by *dfrC* (98.95%), *PC1_blaZ* (84.21%), and *mecA* (78.42%). In contrast, the detection rates of several resistance genes (such as *qacB*, *TEM-116*, *Lreu_cat-TC*, and *FosB1*) were relatively low. When comparing the carriage rates of genes for resistance between homologous and non-homologous isolates (Table 2), there were significant differences in two genes after false discovery rate (FDR) correction for multiple comparisons, with a high carriage rate of the tetracycline-resistance gene *tet(K)* (35.90% vs 15.89%; adjusted *P* = 0.032) and the aminoglycoside-resistance gene *ANT(4′)-Ib* (38.46% vs 18.54%; adjusted *P* = 0.032) in homologous isolates.

**TABLE 2:** Resistance genes of homologous and non-homologous MRSE isolates^*b*^^,*c*^

Resistance genes (positive)	MRSE isolates (*n* = 190)	Homologous isolates (*n* = 39)	Non-homologous isolates (*n* = 151)	Unadjusted *P-*value	Adjusted *P*-value
Tetracycline					
*tet(K*)	38 (20.00)	14 (35.90)	24 (15.89)	0.005	**0.032**
*tet(L*)	2 (1.05)	0 (0.00)	2 (1.32)	0.631^*a*^	0.203
Fluoroquinolone					
*qacA*	74 (38.95)	18 (46.15)	56 (37.09)	0.301	0.203
*qacB*	1 (0.53)	0 (0.00)	1 (0.66)	0.795^*a*^	0.203
Multidrug					
*mgrA*	190 (100.00)	39 (100.00)	151 (100.00)	–^*d*^	–
*norA*	190 (100.00)	39 (100.00)	151 (100.00)	–	–
Aminoglycoside					
*AAC6_Ie_APH2_Ia*	31 (16.32)	8 (20.51)	23 (15.23)	0.283^*a*^	0.203
*APH(3′)-IIIa*	12 (6.32)	4 (10.26)	8 (5.30)	0.214^*a*^	0.203
*ANT(4′)-Ib*	43 (22.63)	15 (38.46)	28 (18.54)	0.009	**0.032**
Beta-lactam					
*PC1_blaZ*	160 (84.21)	32 (82.05)	128 (84.77)	0.672	0.203
*mecI*	3 (1.58)	0 (0.00)	3 (1.99)	0.500^*a*^	0.203
*mecR1*	3 (1.58)	0 (0.00)	3 (1.99)	0.500^*a*^	0.203
*mecA*	149 (78.42)	34 (87.18)	115 (76.16)	0.136	0.203
*TEM-116*	1 (0.53)	0 (0.00)	1 (0.66)	0.795^*a*^	0.203
Macrolides/streptogramin/lincosamides					
*mphC*	73 (38.42)	14 (35.90)	59 (39.07)	0.716	0.203
*lnuA*	43 (22.63)	8 (20.51)	35 (23.18)	0.723	0.203
*ermC*	56 (29.47)	8 (20.51)	48 (31.79)	0.169	0.203
*msrA*	75 (39.47)	14 (35.90)	61 (40.40)	0.608	0.203
*vgaALC*	5 (2.63)	0 (0.00)	5 (3.31)	0.453	0.203
*vgaA*	27 (14.21)	7 (17.95)	20 (13.25)	0.267	0.203
Streptothricin					
*SAT-4*	3 (1.58)	1 (2.56)	2 (1.32)	0.500^*a*^	0.203
Phenol					
*Efac_ACT_CHL*	2 (1.05)	0 (0.00)	2 (1.32)	0.631^*a*^	0.203
*Lreu_cat-TC*	1 (0.53)	0 (0.00)	1 (0.66)	0.795^*a*^	0.203
*Sint_ACT_CHL*	20 (10.53)	4 (10.26)	16 (10.60)	0.608^*a*^	0.203
*catA8*	4 (2.11)	1 (2.56)	3 (1.99)	0.604^*a*^	0.203
Fosfomycin					
*FosB1*	1 (0.53)	0 (0.00)	1 (0.66)	0.795^*a*^	0.203
Fusidic acid					
*fusB*	29 (15.26)	4 (10.26)	25 (16.56)	0.240	0.203
*fusC*	2 (1.05)	0 (0.00)	2 (1.32)	0.631^*a*^	0.203
Sulfonamides					
*dfrC*	188 (98.95)	38 (97.44)	150 (99.34)	0.369^*a*^	0.203
*dfrG*	54 (28.42)	6 (15.38)	48 (31.79)	0.043	0.102

^*a*^ Estimated by the Fisher’s exact probability method.

^*b*^ Data are presented as no. (%) or as otherwise indicated. Boldface indicates statistical significance.

^*c*^ MRSE, methicillin-resistant *S. epidermidis*.

^*d*^ –, not applicable.

**Fig 2:**
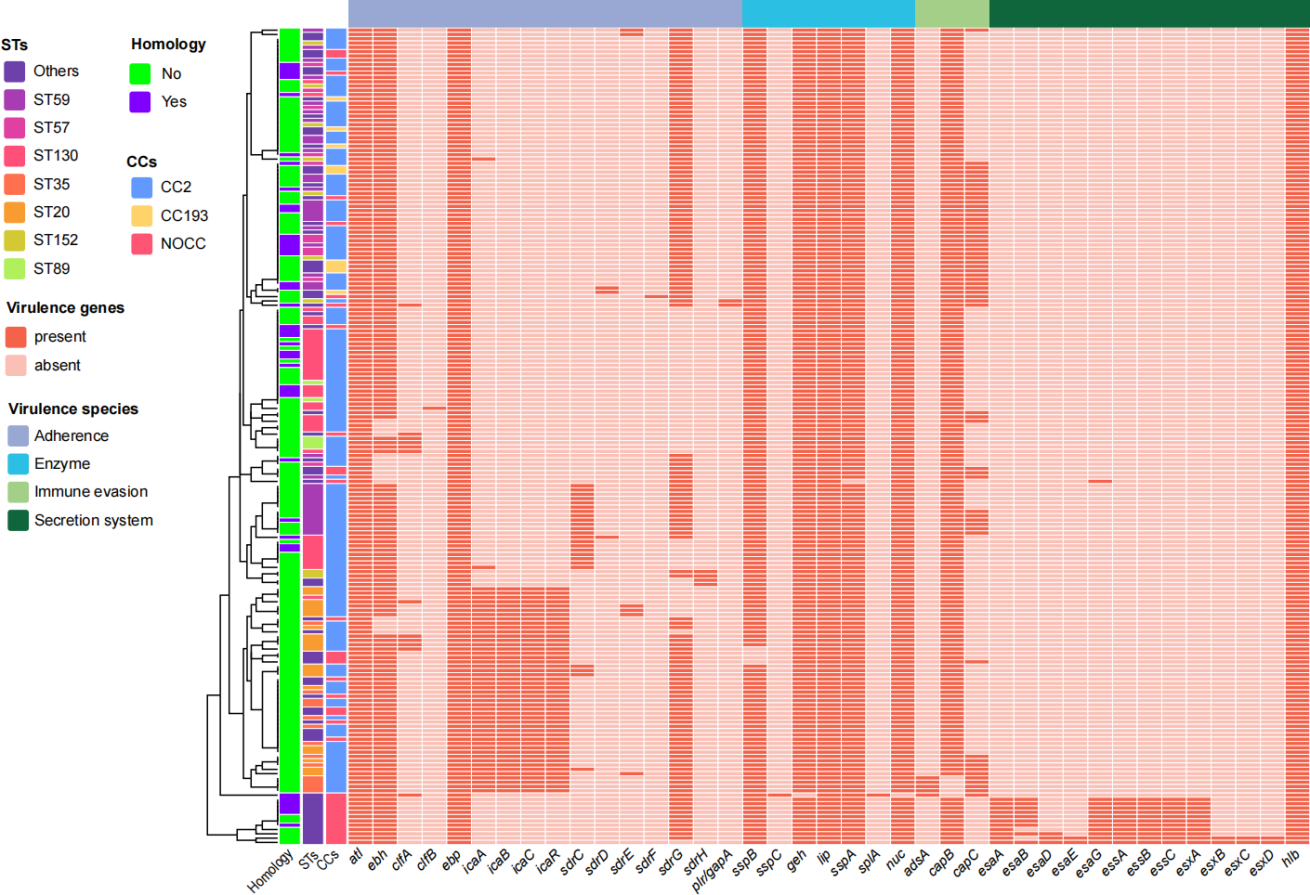
Virulence genes of homologous and non-homologous MRSE isolates.

A total of 38 genes for virulence were identified, including virulence genes associated with adhesion, enzyme, immune evasion, and secretory system. The main virulence factors were adhesion-associated genes, which accounted for 39.47% (15/38; Fig. 2). As to the adhesion-associated genes, the most prevalent genes were *atl* and *ebp* (100%), followed by *ebh* (92.11%) and *sdrG* (72.63%). Among the enzyme-associated genes, the predominant gene was the *lip* (100%), followed by *geh* (99.47%), *sspA* (99.47%), *nuc* (99.47%), and *sspB* (97.89%). For the immune evasion genes, *capB* was detected in 97.37%, *capC* in 30.53%, and *adsA* in 2.63%. With regard to genes associated with the secretory system, only the *hlb* gene was present in all MRSE isolates, while the *esa*, *ess*, and *esx* gene clusters were detected in less than 10%. When comparing the carriage rates of genes for virulence between homologous and non-homologous isolates (Table 3), there were significant differences in several genes for virulence after FDR correction for multiple comparisons, with a high carriage rate of the secretion-associated genes (*esaB*, *essA*, *essB*, *essC*, and *esxA*; all adjusted *P* < 0.050) but low carriage rates of adhesion-associated genes (*icaA*, *icaB*, *icaC*, and *icaR*; all adjusted *P* = 0.002) and secretion-associated gene *esaA* (adjusted *P* = 0.041) in homologous isolates.

**TABLE 3:** Virulence genes of homologous and non-homologous MRSE isolates^*b*^^,*c*^

Virulence genes (positive)	MRSE isolates (*n* = 190)	Homologous isolates (*n* = 39)	Non-homologous isolates (*n* = 151)	Unadjusted *P-*value	Adjusted *P*-value
Adherence					
*atl*	190 (100.00)	39 (100.00)	151 (100.00)	_	_^*d*^
*ebh*	175 (92.11)	8 (97.44)	137 (2.56)	0.144^*a*^	0.092
*clfA*	12 (6.32)	2 (6.62)	10 (5.13)	0.537^*a*^	0.168
*clfB*	1 (0.53)	1 (0.66)	0 (0.00)	0.795^*a*^	0.193
*ebp*	190 (100.00)	39 (100.00)	151 (100.00)	_	_
*icaA*	50 (26.32)	0 (0.00)	50 (33.11)	<0.001^*a*^	**0.002**
*icaB*	48 (25.26)	0 (0.00)	48 (31.79)	<0.001^*a*^	**0.002**
*icaC*	48 (25.26)	0 (0.00)	48 (31.79)	<0.001^*a*^	**0.002**
*icaR*	48 (25.26)	0 (0.00)	48 (31.79)	<0.001^*a*^	**0.002**
*sdrC*	24 (12.63)	4 (10.26)	20 (13.25)	0.425^*a*^	0.146
*sdrD*	3 (1.58)	2 (5.13)	1 (0.66)	0.108^*a*^	0.074
*sdrE*	6 (3.16)	0 (0.00)	6 (3.97)	0.247^*a*^	0.113
*sdrF*	1 (0.53)	0 (0.00)	1 (0.66)	0.795^*a*^	0.193
*sdrG*	138 (72.63)	27 (69.23)	111 (73.51)	0.593	0.672
*sdrH*	4 (2.11)	0 (0.00)	4 (2.65)	0.396^*a*^	0.146
*plr/gapA*	2 (1.05)	1 (2.56)	1 (0.66)	0.369^*a*^	0.146
Enzyme					
* sspB*	186 (97.89)	39 (100.00)	147 (97.35)	0.396^*a*^	0.146
*sspC*	1 (0.53)	1 (2.56)	0 (0.00)	0.205^*a*^	0.100
*geh*	189 (99.47)	38 (97.44)	151 (100.00)	0.205^*a*^	0.100
*lip*	190 (100.00)	39 (100.00)	151 (100.00)	_	_
*sspA*	189 (99.47)	39 (100.00)	150 (99.34)	0.795^*a*^	0.193
*splA*	1 (0.53)	1 (2.56)	0 (0.00)	0.205^*a*^	0.100
*nuc*	189 (99.47)	38 (97.44)	151 (100.00)	0.205^*a*^	0.100
Immune evasion					
*adsA*	5 (2.63)	1 (2.56)	4 (2.65)	0.727^*a*^	0.193
*capB*	185 (97.37)	38 (97.44)	147 (97.35)	0.727^*a*^	0.193
*capC*	58 (30.53)	44 (29.14)	14 (35.90)	0.264^*a*^	0.115
Secretion system					
*esaA*	11 (5.79)	6 (3.97)	5 (12.82)	0.050^*a*^	**0.041**
*esaB*	9 (4.74)	5 (12.82)	4 (2.65)	0.019^*a*^	**0.031**
*esaD*	3 (1.58)	0 (0.00)	3 (1.99)	0.500^*a*^	0.146
*esaE*	2 (1.05)	0 (0.00)	2 (1.32)	0.631^*a*^	0.168
*esaG*	12 (6.32)	5 (12.82)	7 (4.64)	0.073^*a*^	0.055
*essA*	11 (5.79)	5 (12.82)	6 (3.97)	0.050^*a*^	**0.041**
*essB*	11 (5.79)	5 (12.82)	6 (3.97)	0.050^*a*^	**0.041**
*essC*	11 (5.79)	5 (12.82)	6 (3.97)	0.050^*a*^	**0.041**
*esxA*	11 (5.79)	5 (12.82)	6 (3.97)	0.050^*a*^	**0.041**
*esxB*	2 (1.05)	0 (0.00)	2 (1.32)	0.631^*a*^	0.168
*esxC*	2 (1.05)	0 (0.00)	2 (1.32)	0.631^*a*^	0.168
*esxD*	2 (1.05)	1 (0.68)	1 (2.27)	0.410^*a*^	0.146
*hlb*	190 (100.00)	39 (100.00)	151 (100.00)	_	_

^*a*^ Estimated by the Fisher’s exact probability method.

^*b*^ Data are presented as no. (%) or as otherwise indicated. Boldface indicates statistical significance.

^*c*^ MRSE, methicillin-resistant *S. epidermidis.*

^*d*^ –, not applicable.

### Molecular typing of homologous and non-homologous MRSE isolates

In terms of sequence types (STs, the most common genotypes for MRSE isolates were ST59 (37 isolates, 19.47%) and ST130 (36 isolates, 18.95%), followed by ST20 (20 isolates, 10.53%), ST35 (13 isolates, 6.84%), and ST57 (11 isolates, 5.79%). Additionally, six new STs were discovered and designated as ST1202 to ST1207, which have been uploaded on PubMLST. By performing e-BURST (goeBURST) analysis, we identified two clonal complex (CC) types, namely, CC2 and CC193. Among them, CC2 is the most prevalent clone (147 isolates, 77.37%), with ST2 as the origin and a total of 18 STs; and CC193 (nine isolates, 4.74%) has a total of 4 STs. There were statistically significant differences in the proportion of specific STs (ST130, ST20, ST35, and ST57) between homologous and non-homologous isolates (all *P* < 0.05, Table 4), with ST130 and ST57 being significantly higher proportion in homologous isolates.

**TABLE 4:** Predominant genotypes of homologous and non-homologous MRSE isolates^*b*^^,^*^c^*

Genotypes	MRSE isolates (*n* = 190)	Homologous isolates (*n* = 39)	Non-homologous isolates (*n* = 151)	*P*-value
STs				
ST59	37 (19.47)	8 (20.51)	29 (19.21)	0.854
ST130	36 (18.95)	13 (33.33)	23 (15.23)	**0.010**
ST20	20 (10.53)	0 (0.00)	20 (13.25)	**0.008^*a*^**
ST35	13 (6.84)	0 (0.00)	13 (8.61)	**0.045^*a*^**
ST57	11 (5.79)	7 (17.95)	4 (2.65)	**<0.001**
CCs				
CC2	147 (77.37)	30 (76.92)	117 (77.48)	0.941
CC193	9 (4.74)	0 (0.00)	9 (5.96)	0.120^*a*^

^*a*^ Estimated by the Fisher’s exact probability method.

^*b*^ Data are presented as no. (%) or as otherwise indicated. Boldface indicates statistical significance.

^*c*^ MRSE, methicillin-resistant *S. epidermidis*; ST, sequence type; CC, clone complex.

### Predicting homologous MRSE isolates with high transmission risk

**Fig 3:**
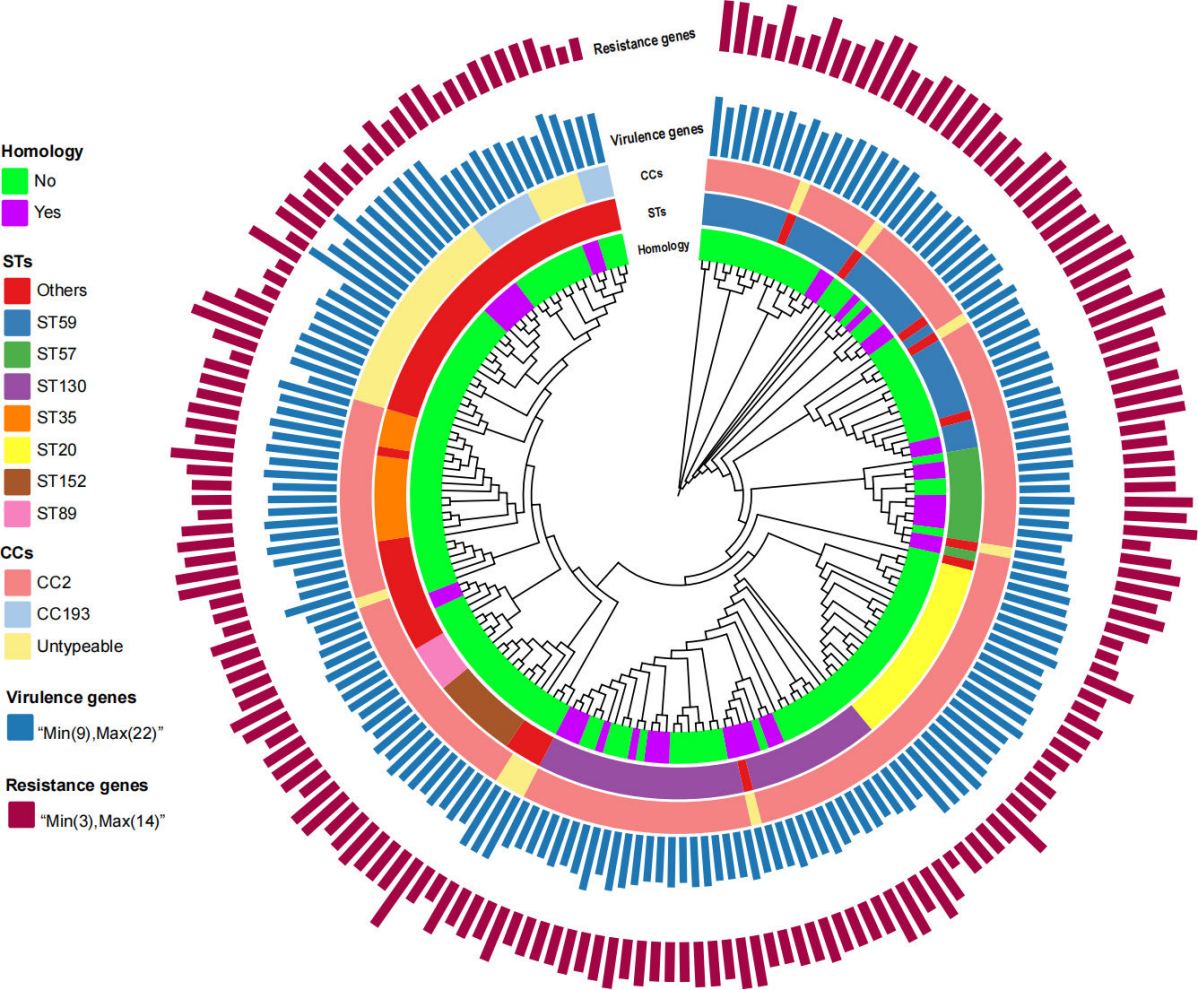
Phylogenetic tree showing the genetic similarity of 190 MRSE isolates. The colored bands at the top of the tree (from the inside to the outside) represent the homology, CC, sequence type, the number of resistance genes, and the number of virulence genes.

**Fig 4:**
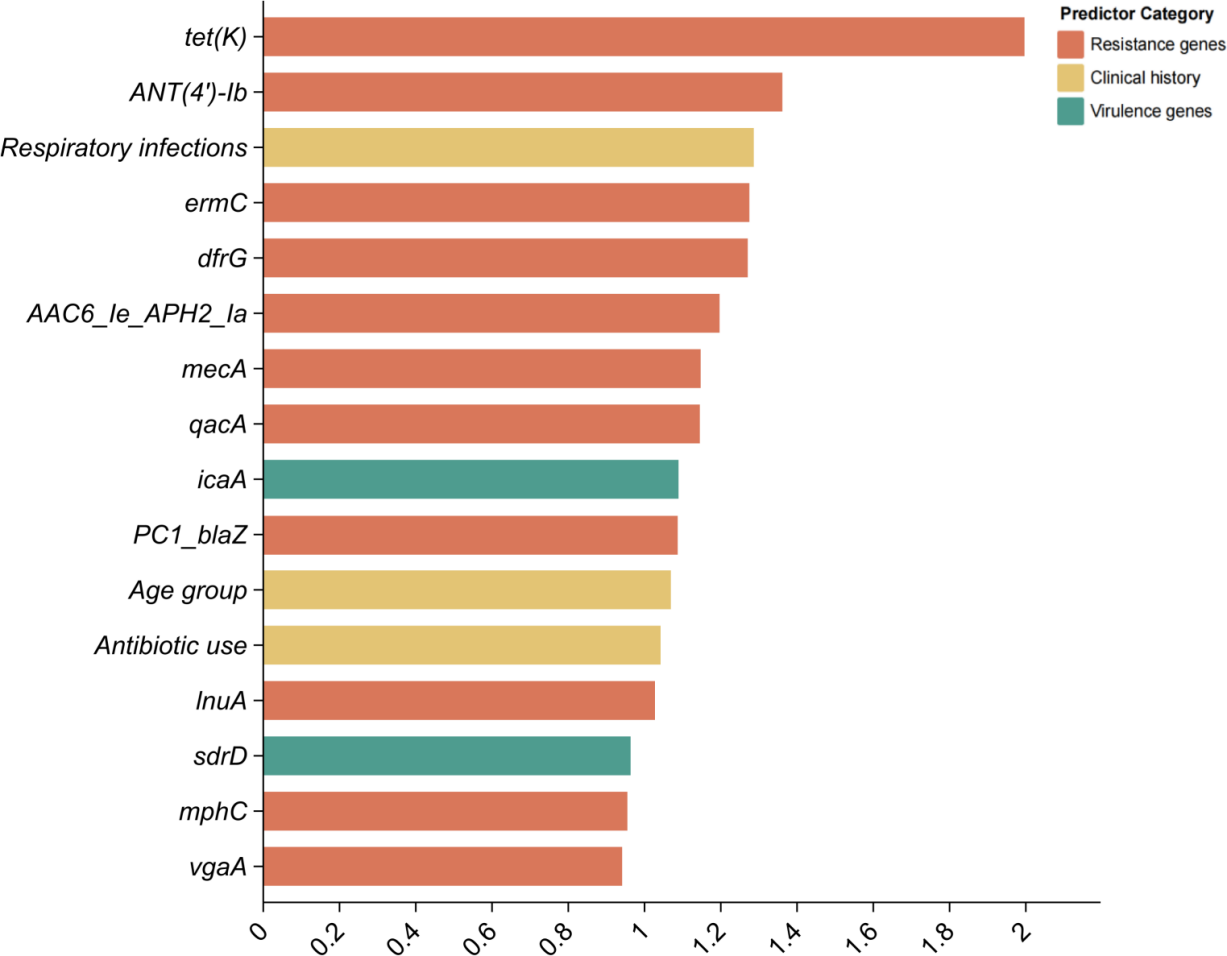
Variable importance of predictor factors in the Random Forest model.

According to Fig. 3 and Tables 2 to 4, we observed that there are significant differences in molecular characteristics (resistance and virulence genes) between homologous and non-homologous isolates. The Random Forest method was employed to predict homologous MRSE isolates with high transmission risk based on resistance and virulence genes. The final model achieved a classification accuracy of 81.58% (95% confidence interval [CI]: 65.67% to 92.26%), with a sensitivity of 71.43% (95% CI: 29.04% to 96.33%), a specificity of 83.87% (95% CI: 66.27% to 94.55%), and an area under the curve value of 0.89 (95% CI: 0.80 to 0.99). These results indicate that this model has stable predictive performance and also exhibits high classification accuracy in predicting homologous isolates with high transmission risk. The importance of predictor factors is shown in Fig. 4, suggesting that the top five highest-ranked factors associated with MRSE transmission were *tet(K*), *ANT(4′)-Ib,* respiratory infections, *ermC*, and *dfrG*.

### Inference of potential transmission clusters and routes of MRSE isolates

**Fig 5:**
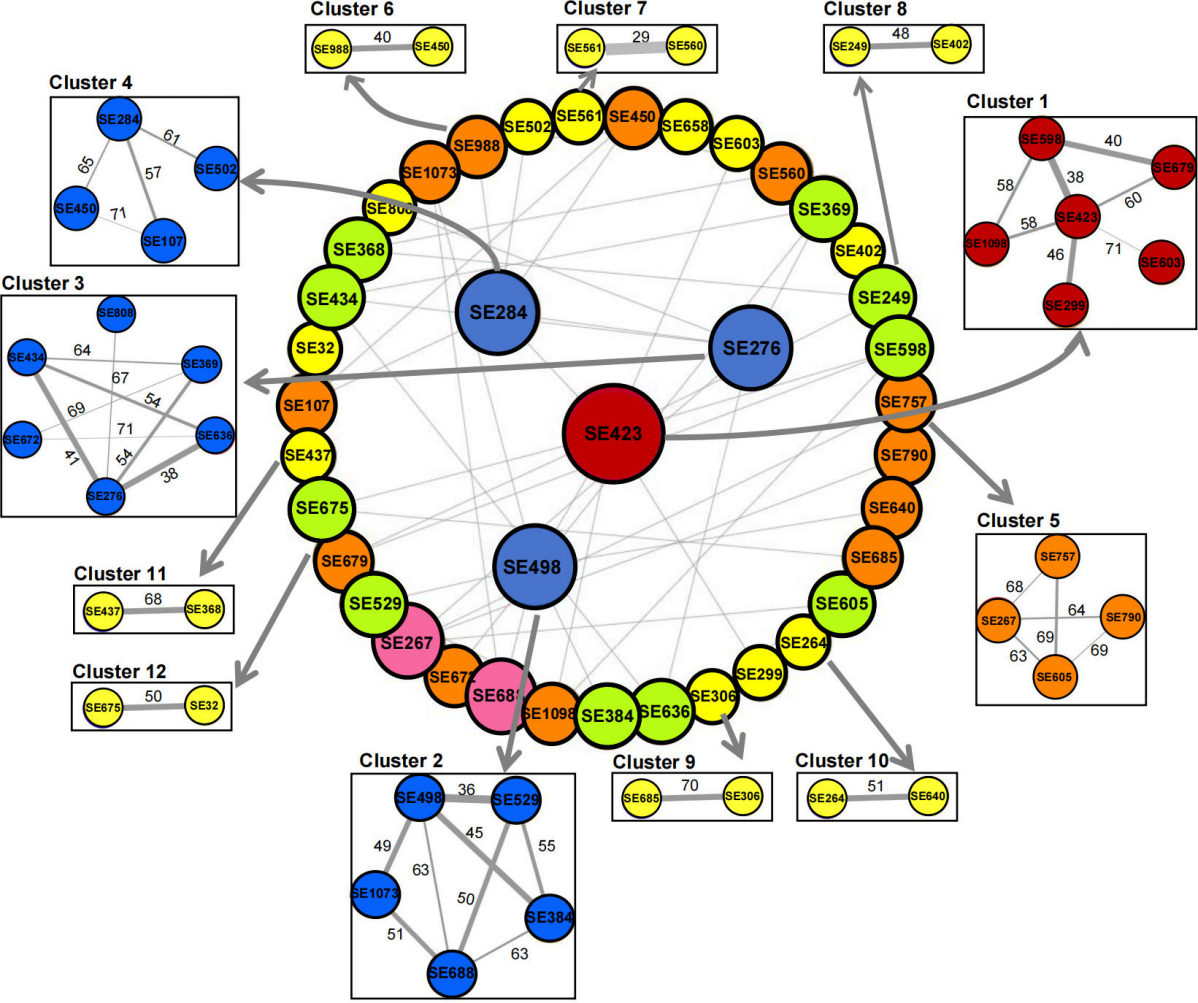
Putative transmission clusters of homologous MRSE isolates. The central network represents the transmission network of all homologous strains constructed based on SNP distances (threshold ≤ 71), with 12 transmission clusters identified. Each node represents an isolate. The numbers on the connecting lines indicate the SNP distance between isolates. The thickness of the lines represents the SNP distance, with thicker lines for smaller SNP differences. The color of the circles is distinguished based on the number of homologous isolates.

According to the homology analysis of MRSE isolates with a genomic difference of less than 71 SNPs, we inferred the potential transmission clusters and routes, with 12 homologous transmission clusters (two large clusters, three medium clusters, and seven small clusters) and 40 transmission routes identified (Fig. 5). To assess the genetic relatedness of MRSE isolates within each cluster, we calculated the pairwise SNP divergence between non-repetitive isolates. This distribution of these SNP distances suggests that homologous isolates may have undergone similar genetic changes during the evolutionary process. According to the phylogenetic tree, we observed three largest clusters (cluster 1, cluster 2, and cluster 3). In these transmission clusters, the cluster 1 with six homologous MRSE isolates formed seven homologous transmission routes, the cluster 2 with five homologous isolates had eight transmission routes, and the cluster 3 with six homologous isolates had eight homologous transmission routes, indicating that these transmission events may occur between multiple sexual partners. Notably, most isolates from the biggest transmission clusters belonged to ST57 and also carried the *tet(K*) gene, providing important genetic evidence for identifying high-risk genotypes. In contrast, there were seven smallest clusters (SE437, SE264, SE249, SE32, SE658, SE685, and SE560), and each cluster had only two isolates, suggesting that most transmission events may occur between two sexual partners. Detailed transmission routes for each cluster were provided in Table 5. The above results offer a detailed and comprehensive view of the distribution and genetic differences between homologous MRSE isolates, highlighting the complex dynamics of MRSE transmission among MSM.

**TABLE 5:** Transmission clusters and transmission routes of MRSE isolates^*a*^

Clusters	Number of homologous isolates	Homologous transmission routes
cluster 1	6	SE598↔SE1098, SE598↔SE679, SE598↔SE423, SE423↔SE1098, SE423↔SE679, SE423↔SE299, SE423↔SE603
cluster 2	5	SE598↔SE529, SE498↔SE384, SE498↔SE688, SE498↔SE1073, SE529↔SE384, SE529↔SE688, SE384↔SE688, SE688↔SE1073
cluster 3	6	SE276↔SE636, SE276↔SE369, SE276↔SE808, SE276↔SE434, SE636↔SE434, SE636↔SE672, SE369↔SE434, SE369↔SE672
cluster 4	4	SE284↔SE502, SE284↔SE450, SE284↔SE107, SE450↔SE107
cluster 5	4	SE267↔SE605, SE267↔SE790, SE267↔SE757, SE605↔SE790, SE605↔SE757
cluster 6	2	SE988↔SE450
cluster 7	2	SE560↔SE561
cluster 8	2	SE249↔SE402
cluster 9	2	SE685↔SE306
cluster 10	2	SE264↔SE640
cluster 11	2	SE437↔SE368
cluster 12	2	SE32↔SE675

^*a*^ MRSE, methicillin-resistant *S. epidermidis*.

## DISCUSSION

*S. epidermidis* is recognized as one of the predominant commensal bacteria residing in the human nasal cavity (16). Importantly, MRSE is a multidrug-resistant pathogen causing infections associated with prolonged hospitalization, high morbidity, and high mortality (17). Our findings revealed a high carriage rate of *S. epidermidis* (82.75%) and MRSE (44.12%), which were significantly higher than results from Sudanese healthy individuals (24.09% for *S. epidermidis*) (18), Japanese healthy children (19.30% for MRSE) (19), the community population in Portugal (54.00% for *S. epidermidis* and 7.00% for MRSE) (6), healthy population in Southern China (34.89% for *S. epidermidis* and 14.08% for MRSE) (20), and the community population in Eastern China (44.80% for *S. epidermidis* and 17.20% for MRSE) (7), but lower than that of infected neonates in China (52.00% for MRSE) (21). There may be several reasons for this difference, such as host factors (e.g., antimicrobial use, infection history, hospitalization, and animal contact), sampling methods, bacterial identification methods, and geographical location. Importantly, in this study, the nasal carriage rates of *S. epidermidis* and MRSE in the MSM population were significantly higher than those reported in both healthy and infected populations, indicating that the MSM population is at a high risk of MRSE infection and also serves as an important source of community transmission.

The antimicrobial resistance of MRSE has become an important public health issue. Notably, in this study, MRSE exhibited high rates of resistance to multiple antimicrobials, particularly for penicillin (93.68%), cefoxitin (85.26%), and erythromycin (75.79%), which is associated with the imprudent use of penicillin in China. These findings are consistent with reports from Portugal, the United Kingdom, and Sweden (6, 11, 22). Vancomycin and linezolid were regarded as the first-line antimicrobials for serious bacterial infection or sepsis. Importantly, in this study, all MRSE isolates were susceptible to vancomycin and rifampicin, and most of the MRSE isolates were susceptible to linezolid, which may be attributed to the stringent control measures regarding vancomycin and linezolid use in clinical settings. This study identified several resistance and virulence genes. Among these resistance genes, all MRSE isolates carried the multidrug-resistant genes *mgrA* and *norA*, followed by the sulfonamide resistance-associated gene *dfrC* and the beta-lactam resistance-associated gene *PC1_blaZ* and *mecA*, suggesting that these genes are widely prevalent in MRSE isolates. Similarly, a previous study in Saudi Arabia found that all methicillin-resistant *S. aureus* possessed *mecA*, *mgrA*, *norA*, and *PC1_blaZ* (23). Due to the absence of coagulase, *S. epidermidis* is generally regarded as possessing fewer virulence genes and lower pathogenicity compared to *S. aureus* (24). Among the adhesion factors, the most prevalent genes were *atl*, *ebp*, *ebh*, and *sdrG* genes, which are consistent with the results from South Africa (25). Identifying potential transmission risk of MRSE is the first step for preventing and controlling transmission. In our exploratory analysis, the resistance genes *tet(K*) and *ANT(4′-Ib* were significantly higher in homologous isolates than in non-homologous isolates. In addition, the final prediction model based on key predictors, including *tet(K*) and *ANT(4′)-Ib*, achieved a high classification accuracy of 81.58%, offering a simple model for identifying the super-spreading MRSE isolates after further functional validation. We observed that homologous MRSE isolates exhibited a distinct resistance profile, with significantly higher resistance to cefoxitin but lower resistance to erythromycin compared to non-homologous isolates. This resistance profile may be attributed to the fitness trade-off associated with carrying the *mecA* gene, and the acquisition and maintenance of *mecA* and its surrounding *SCCmec* elements often impose significant metabolic burden (26–28). Loss of accessory resistance genes (e.g., conferring erythromycin resistance) may compensate for this burden and increase fitness. Furthermore, all homologous isolates lacked the *ica* genes (e.g., *icaA*, *icaC*, and *icaR*). This reflects the fitness trade-off, in which the transmissibility through planktonic growth is achieved at the expense of persistent biofilm formation (29).

Considering the specificity of the MSM population, it is urgently needed to clarify potential transmission dynamics of MRSE. The phylogenetic tree and homology analysis identified 12 transmission clusters and 40 transmission routes of homologous MRSE isolates. The largest clusters (cluster 1, cluster 2, and cluster 3) containing five to six homologous MRSE isolates and seven to eight transmission routes revealed that these homologous transmission events may occur between multiple sexual partners, providing valuable insights for identifying risk genotypes of the super bacteria, while the smallest clusters with only two homologous isolates suggested that their transmission events may occur between two sexual partners. These findings highlight the complex dynamics of MRSE transmission among MSM. MRSE transmission between MSM may occur through close contact methods such as oral intercourse, anal intercourse, and other contact routes. Note that most isolates from the biggest transmission clusters belonged to ST57 and also carried the *tet(K*) gene, providing important genomic determinants for identifying risk genotypes of super-spreaders. However, it lacked epidemiological information to ascertain the transmission direction. Future prospective cohort studies will need to trace the transmission directions and routes of MRSE. In this study, several predominant STs were identified, including ST59, ST130, ST20, ST35, and ST57. The distribution of these STs had significant differences between homologous and non-homologous isolates, with significantly high rates of ST130 and ST57 in homologous isolates. This suggests that specific STs may be linked to the transmission clusters of MRSE. For instance, ST130 and ST57 may be transmitted within MSM communities through close contact (such as sexual contact). Compared to the previous study (30), this study identified several novel genotypes of MRSE (such as ST1202-ST1207) within the MSM population, indicating that MRSE may exhibit genetic diversity across different populations.

This study provides important insights into molecular characteristics and transmission dynamics of MRSE among the MSM population and also offers a simple model for identifying the transmission risk of MRSE in the community setting. However, there are several potential limitations. Firstly, this study is a cross-sectional design monitored at only one time point, so we could not determine whether the MRSE carriage among participants was transient or persistent. This limitation can be overcome by future multi-stage longitudinal studies to reveal potential colonization persistence and transmission dynamics. Secondly, *S. epidermidis* is the most abundant species in the microbiota of the human nasal cavity, so this study primarily focuses on nasal swab samples, which may underestimate the true prevalence by missing extra-nasal sites (e.g., the rectum). However, nasal sampling as a practical approach for community surveillance has been widely used in previous studies for healthy populations (1), and our results were consistent with previous reports. For high-risk populations such as MSM, future expanding sampling in other anatomical sites (such as the rectum or urethra) would improve ecological validity. Third, this study inferred potential transmission clusters based on SNP differences but lacked epidemiological contact data to ascertain the transmission direction. Notably, in privacy-sensitive populations like MSM where stigma and legal risks may impede self-reported sexual behaviors or contact network data, our SNP-based method provides an objective, non-invasive strategy to identify high-risk transmission clusters for targeted interventions. Finally, the small sample size of homologous isolates (*n* = 39) may have limited the power of revealing the potential transmission routes. So future large-sample cohort studies should be performed to elucidate the detailed transmission mechanisms.

## MATERIALS AND METHODS

### Study participants

This cross-sectional study was conducted from April to August 2019 in Guangzhou City, China. Study participants were MSM above 18 years of age who had penetrative oral or anal intercourse with other men within the past year. Convenience sampling was used to enroll participants at the HIV Voluntary Counseling and Testing clinic of the Lingnan Partner Community Support Center and the Guangzhou Municipal Center for Disease Prevention and Control. The eligibility criteria for study participants included (i) being above 18 years old, (ii) having penetrative oral or anal intercourse with other men within the past year, and (iii) obtaining informed consent. In all, there were 510 MSM sampled in this study.

### Sample size estimation

The sample size for this study was determined using the formula for sample size calculations for prevalence studies (31). The standard normal variate was set at 5% type I error, the precision was set at 0.95, and the prevalence of MRSE was 10.6% (32). The minimum sample size was 145 for MRSE with power of 50%. In this study, we obtained a total of 190 MRSE isolates for whole-genome sequencing analysis.

### Identification of bacterial isolates

After obtaining informed consent, two nasal swabs were taken from each participant. The collected samples were subjected to enrichment culture and subsequent testing, including Gram staining microscopy, catalase test, hemolysis test, and plasma coagulase test. Based on the above results, the isolates were preliminarily identified as coagulase-negative *Staphylococcus*. Then, PCR assays were performed to test the *epi* and *mecA* genes (33). If the *epi* was positive, the isolates were identified as *S. epidermidis*. If the *mecA* was positive, the *S. epidermidis* isolates were identified as MRSE (33).

### Antimicrobial susceptibility testing

According to the guidelines of the Clinical and Laboratory Standards Institute 2022, antimicrobial susceptibility testing was conducted on 13 commonly used antimicrobials (penicillin, cefoxitin, erythromycin, tetracycline, co-trimoxazole, gentamicin, teicoplanin, clindamycin, chloramphenicol, levofloxacin, rifampicin, vancomycin, and linezolid) using the Kirby-Bauer disc diffusion method. The measurement of the inhibition zone diameter is used to determine resistance to the aforementioned antimicrobials.

### Whole-Genome sequencing

Genomic DNA of all MRSE isolates was extracted using the Magen Hipure Bacterial DNA Kit, following manufacturer’s instructions. Then, high-throughput whole-genome sequencing (WGS) was performed using the Illumina HiSeq 2000 machine (Illumina, https://www.illumina.com/), obtaining paired-end 150 bp reads. The quality of the raw sequenced reads was assessed using FastQC version 0.11.9 (https://github.com/s-andrews/FastQC). Taxonomic classification and identification of low-level contamination were checked by Kraken 2 (https://github.com/DerrickWood/kraken2). The raw reads were assembled using SPAdes version 3.6.1 (https://github.com/ablab/spades), with read error correction enabled. We used the pubMLST database of *S. epidermidis* to predict the ST of each isolate based on seven housekeeping genes (*arc*, *aroE*, *gtr*, *mutS*, *pyrR*, *tpiA*, and *yqiL*) and adopted the eBURST algorithm to infer the CC. Antimicrobial-resistance genes were determined using the Comprehensive Antibiotic Resistance Database (https://card.mcmaster.ca), and virulence genes were identified using the Virulence Factors databases (https://www.mgc.ac.cn/VFs/).

### Phylogenetic analysis and homology analysis

The maximum-likelihood phylogenetic tree was constructed using the core genome SNPs identified by kSNP3.0 software. The phylogeny was annotated and visualized in ChiPlot (https://www.chiplot.online). Additionally, the differences in SNPs among strains and the number of SNPs for each pairwise comparison were analyzed. The kSNPdist software was used to calculate the pairwise SNP divergence between non-repetitive isolates. By comparing the SNP distances between different isolates, we gained insights into their genetic similarities. The kSNP3 and kSNPdist are freely available at https://sourceforge.net/projects/ksnp/.

In the homology analysis, the homology threshold of MRSE isolates was set at 71 SNPs, with a SNP distance of less than 71 between two isolates defined as homologous isolates (34). Genomic-based transmission clusters of MRSE isolates were identified relying on homologous isolates with a genomic difference of less than 71 SNPs. The size of a transmission cluster was determined based on the total number of genomes it encompassed, categorized as small (two genomes), medium (three to five genomes), or large (>5 genomes). The results were visualized using Cytoscape (35).

### Statistical analysis

We compared the proportions of resistance phenotypes, resistance genes, virulence genes, and genotypes (STs and CCs) between homologous and non-homologous isolates using Pearson’s chi-squared test or Fisher’s exact test. Two-sided *P*-value of <0.05 was defined as being of statistical significance. The Benjamini-Hochberg FDR was used to correct for false-positive rates due to multiple hypothesis testing. These statistical analyses were conducted using Stata V16.0 (StataCorp LP, College Station, Texas, USA). Random Forest is an ensemble learning method based on decision trees, which has been widely adopted for handling high-dimensional data. Considering high-correlated genomic data, we performed the Random Forest model for risk prediction by the R package “randomForest,” using MRSE types (homologous or non-homologous) as outcome variable and potential predictors (resistance genes and virulence genes) as independent variables. We also incorporated several important confounding variables into the Random Forest model, including age strata (≤27 or >27 years), infection history in the last 6 months (HIV, syphilis, and respiratory infections), and antibiotic use in the last 6 months.

### Conclusion

This study revealed high nasal carriage rates of 82.75% for *S. epidermidis* and 44.12% for MRSE among MSM, with unique molecular profiles potentially associated with transmission clusters, emphasizing the MSM population as a crucial reservoir for MRSE. The genomic analysis of MRSE indicated 20.53% homologous isolates, forming 12 transmission clusters and 40 transmission routes. These findings highlight the complex transmission dynamics of MRSE in this MSM population. In addition, the Random Forest model achieved an accuracy of 81.58% in predicting homologous transmission risk based on resistance and virulence biomarkers. The integration of targeted screening with genomic surveillance could significantly prevent the spread of MRSE in the high-risk MSM population.

## Data Availability

The sequence data have been submitted to Sequence Read Archive database under PRJNA1283353.
